# The Moderation Effects of Comparative Thinking Between Gratitude and Negative Affect During the COVID-19 Outbreak

**DOI:** 10.3389/fpsyg.2021.644323

**Published:** 2021-08-23

**Authors:** Gloria Bernabe-Valero, José Salvador Blasco-Magraner, Remedios Aguilar-Moya, Carmen Moret-Tatay

**Affiliations:** ^1^MEB Lab (Mind, Emotion, and Behavior Research Laboratory), Catholic University of Valencia San Vicente Mártir, Valencia, Spain; ^2^Facultad de Magisterio, Universidad de Valencia, Valencia, Spain; ^3^Facultad de Magisterio y Ciencias de la Educación, Universidad Católica de Valencia San Vicente Mártir, Valencia, Spain; ^4^Dipartimento di Neuroscienze Salute Mentale e Organi di Senso (NESMOS), Sapienza Università di Roma, Rome, Italy

**Keywords:** COVID-19, positive affect, negative affect, gratitude, gender

## Abstract

The aim of this research was to examine the moderation effects of comparative thinking (CT) across the relationship between gratitude and affect during the COVID-19 outbreak. To this purpose, multiple regression as well as moderation analyses were carried out. Age and sex were also addressed as variables of interest as described in previous literature. A sample of 306 north Americans was recruited by crowdsourcing platform ProA to obtain a representative sample based on age and gender. The participants filled in a questionnaire based on comparative thinking in relation to the emotional experience experienced before and during the COVID-19 outbreak, positive and negative affect schedule for positive and negative affect, as well as Gratitude Questionnaire - Six Items Form scores for gratitude. The main results of the current study related to the COVID-19 outbreak can be listed as follows: (i) no differences between CT groups in the gratitude trait, but differences in positive and negative affect did occur; (ii) regression models that included age, gratitude, and affect variables predicted negative and positive affects but gender did not reach the statistical level; (iii) two moderation models predicted affect from gratitude, with the CT variable moderating this effect; this moderation effect was also statistically significant in predicting negative affect but it was not statistically significant in predicting positive affect. These results might be of interest for training programs in applied levels and theoretical models of gratitude.

## Introduction

Contemporary society has been facing the urgent psychological need for support in an unprecedented health crisis worldwide. This is expected to be followed by an economic crisis of greater impact than that of 2008. Unsurprisingly, this has also raised not only the interest of the scientific community, but also the general public, raising many issues of debate that involve a large body of disciplines in our society. Even though research on this virus has growth exponentially in the last months, there are currently many doubts regarding its mode of transmission and its presentations, in addition to intensive efforts to develop vaccines and therapies. Within this scenario, priority has been given to the biomedical aspect. However, the global psychological impact of this pandemic is still unknown, and merits thoughtful consideration.

Recent studies suggest that the current situation will have a significant psychological impact and an increase on psychological disorders (Duan and Zhu, 2020; Zgueb et al., 2020). A large body of studies has addressed the restriction measures effects in wellbeing and other underlying variables to behavior during lockdown. According to Ammar et al.’s (2020a) findings in Europe, North Africa, West Asia, and America, a psychosocial strain occurs due to large decreases in the amount of social activity through family, friends/neighbors, or entertainment, as well as, lower life satisfaction due to a decrease in mental wellbeing and an increase in depressive symptoms when comparing previous states to confinement (Ammar et al., 2020b). Early studies in China reported that most of the symptoms affecting people in confinement were fear of contagion, anxiety caused by isolation, and lack of information regarding the new virus (Huang and Zhao, 2020). For example, Wang et al. (2020) reported a moderate to severe psychological impact as up to 20% of participants experienced noteworthy depressive symptoms. The role of attitudes towards the COVID-19 outbreak is of interest to describe different profiles in the population that improve adherence to health recommendations (Murphy and Moret-Tatay, 2021). In this way, studies on protective factors seem to be imperative for mental health as well as other inherent fields.

From theoretical models and applied evidence, it has been highlighted how stressful life events can be determinants for many fields (Assari and Lankarani, 2016). Gratitude emerges as a construct of interest in the field of mental health. It has been described as a variable related to happiness, health, purpose in life, and other desirable life outcomes but also related to a decrease in negative affect and vice versa (Rash et al., 2011; Emmons et al., 2019). According to Syropoulos and Markowitz (2020), gratitude is related to various behavioral, affective, and attitudinal responses to the pandemic. Moreover, the authors investigated how this construct is related to moral decisions and response to COVID-19 concluding a positive impact on prosociality. In a previous study, we have found that the four subscales of gratitude (Interpersonal Gratitude, Gratitude in the face of Suffering, Recognition of Gifs, and Expression of Gratitude) were positively associated with positive affect as well as inversely associated with negative affect, indicating that people who are more grateful, both to other people and to transcendental forces, experience a better affective experience (Bernabe-Valero et al., 2021). Moreover, higher scores on gratitude have been also found to be predictors of a lower impact to academic functioning at the end of the semester during the current COVID-19 outbreak (Bono et al., 2020). Thus, we highlight the interest in studying gratitude during the COVID-19 pandemic as it has been found to emerge as a protective factor with numerous benefits for both physical and psychological health (i.e., Emmons and Mishra, 2011) and specifically plays an important role in post-traumatic growth (Linley and Joseph, 2004).

Whether gratitude can be considered a trait or composed effect of states is a subject of much debate in the literature (Wood et al., 2008; Solom et al., 2017). It should be noted that the relationships between the trait of gratitude and daily moods, as well as how this trait behaves in adverse situations such as the current pandemic, are of interest from several perspectives. Not surprisingly, the relationships between the state of gratitude, mood, and traits were investigated from an empirical level, finding that the trait of gratitude was associated with measures of experiences and expressions of gratefulness and appreciation in daily life (e.g., McCullough et al., 2002). Moreover, it was found that measures of gratitude, as an affective trait, are useful for predicting several dimensions of gratitude in people’s daily interpersonal and emotional experience (McCullough et al., 2004). These authors concluded that grateful moods are created both through top-down effects (i.e., the effects of personality and affective traits), bottom-up effects (i.e., the effects of discrete interpersonal and emotional episodes), and the interaction of these effects, providing, in this way, a view of how the three levels of affect affective traits, moods, and emotions are linked (McCullough et al., 2004). With regards to COVID-19, the literature has tried to address to what extent might current experiences explain the relations between traits with general negative appraisal. It was pointed out that situational characteristics often substantially explain the associations of traits with ratings and wellbeing (Kuper et al., 2021).

Another topic that has been addressed in relation to the current pandemic is comparative thinking (e.g., Jahan, 2020). Psychological research has demonstrated how deeply comparisons pervade our thinking (Mussweiler and Epstude, 2009) and the tendency toward comparative information processing is striking because of its remarkable ubiquity. At the time of confinement and the pandemic, many people will have thought about what their life would be like if they were not living in this situation. Moreover, if they could return to the previous state of normality, comparing either their previous life with a standard or imagining their current life without COVID-19. Counterfactual thinking is a type of comparative thinking and can be defined as a process of mentally generating alternatives to a situation (Roese, 1997). Its key feature is the juxtaposition of one’s current status against an imagined better or worse alternative state (Epstude and Roese, 2008). An upward counterfactual is generated when people imagine better alternative states as opposed to a downward counterfactual which is when they imagine worse alternative states (Broomhall et al., 2017). Thus, studies have been found that link gratitude with counterfactual thinking (e.g., McNamara et al., 2003; Nicuța and Constantine, 2021). For example, Teigen (1997) required participants to tell a story of their own regarding two situations in which they had felt grateful and then asked them whether they had thought about what might have happened instead (i.e., whether they had engaged in counterfactual thinking), finding a strong relationship between gratitude and counterfactual thinking. In another study with adolescents, it was found that after engaging in downward comparative thinking, participants reported more gratitude, and levels of negative emotion were decreased (Nicuța and Constantine, 2021). A meta-analysis examined the strength of association between upward counterfactual thinking and depressive symptoms and found that upward counterfactuals and regret produced statistically significant positive effects that were similar in strength and effects. Results also did not vary as a function of the subject related to counterfactual-inducing situations or study designs (in terms of cross-sectional vs. longitudinal), or even different measurement methods (Broomhall et al., 2017). Thus, it is of interest to clarify the role of comparative thinking, both downward and upward, in gratitude and affect. A large body of research has demonstrated that on the level of simple judgments, people engage in comparative thinking (Mussweiler and Epstude, 2009) alluding to comparative judgment as the result of the comparative thinking process. In this study, we are interested in the role of comparative thinking in relation to the emotions and affects experienced in the pandemic.

In this way, we consider that a person’s categorization of their emotional state in a judgment could act as an affective schema that influences their daily affect. In other words, we hypothesize that, for example, if a person summarizes and labels their experience in a predominantly negative way (i.e., indicates that they are *worse* since the pandemic) this will influence their affect, increasing their negativity or reducing their positivity, obtaining different effects if they consider their experience to be *equal* or *better*. Moreover, given that this is comparative thinking, other counterfactual processes may come into play, since a previous situation is compared with the current experience of a pandemic. We therefore consider it interesting to see whether this comparative thinking moderates the existing beneficial effect between gratitude and affectivity. Furthermore, it must be taken into account that some situations that have occurred in the pandemic (such as losses, experiences of uncertainty, among others), have modified individual’s emotional and affective states. In this way, comparisons will necessarily include the result of these experiences, which is a crucial aspect in the field. The aim of this study is to examine the role of comparative thinking regarding COVID-19 on the relationship between gratitude and emotional affect. Age is a variable of interest as differences have been described for gratitude with regard to lifespan (Jiang, 2020). Although the general consensus is that older people exhibit a feeling of greater wellbeing and less negative affectivity in a pandemic (Bernabe-Valero et al., 2021), a research found that older adults showed higher positive affect and lower negative affect in comparison to younger adults, but similar patterns were found for both groups (Ebert et al., 2020). On the other hand, gender differences have been reported on affect during the COVID-19 outbreak in some studies (Terry et al., 2020; Pérez-Mengual et al., 2021) while others (i.e., Cao et al., 2020; Zhang and Ma, 2020) found no differences, reflecting inconsistent results that require further clarification. For this reason, these variables are considered in the current study.

## Materials and Methods

### Participants

Three hundred and six (306) participants were recruited from the prolific platform ProA, with the condition that the entire sample be residents of the United States, whose main language is English. Four participants were excluded because they did not meet this criterion, leaving a sample of 302 participants of which 153 (51%) were women and 149 (49%) were men. A cross-sectional design was used in which ages ranging from 19 to 82years (M=45.07, ST=15.94) were represented. The participants were divided into the following age ranges: 22% between 18 and 29years old, 17% between 30 and 39years old, 15% between 40 and 49years old, 21% between 50 and 59years old, and 25% were 60years and older. Regarding ethnicity: 8% were Asian; 15% Black; 5% were of mixed race, 3% other, and 69% white. As this was a study involving human participants, it was reviewed and approved by the home University Ethical Committee.

### Materials

Before administering the questionnaires, several sociodemographic questions were collected. Specifically, these were related to socio-personal data, age, sex, education level, profession, and employment status.

Comparative thinking in COVID-19 was measured with the question: “*We are currently in a worldwide pandemic situation due to COVID-19. Has this significantly affected your mood and emotions?*” Three answers were possible: “*Yes, I am feeling worse*,” “*No, no change or almost no change*,” “*Yes, I am better*.” This question assessed the participants’ choice about the emotions and affect experienced by comparing the current experience (during the pandemic) and the situation before the pandemic, which is why we call it comparative thinking (CT). We considered that this choice could be the result of a counterfactual thinking process, with an upward counterfactual process occurring when people imagine better alternative states, as opposed to a downward counterfactual process when people imagine worse alternative states. In answering this question, participants chose which response described their state, whether it was upward vs. downward. In order to be able to analyze the comparative thinking, the sample was divided into two levels; the “worse” response group and the “equal or better” one.

Once completed, different measures of gratitude and affect were administered. The instruments are described as follows:

#### Gratitude Questionnaire - Six Items Form

This questionnaire focuses on the emotional component of gratitude (Hudecek et al., 2020) based on an understanding of the concept of gratitude as “a generalized tendency to recognize and respond with grateful emotion to the roles of other people’s benevolence in the positive experiences and outcomes that one obtains” (McCullough et al., 2002, p. 112). The internal consistency of the instrument in its construction was high, being *α*=0.82. It should be noted that item number 6 was removed for theoretical and empirical reasons (see more in Chen et al., 2008; Bernabe-Valero et al., 2013, 2020, 2019; Hudecek et al., 2020). The final GQ-5 internal consistency was *α*=0.89. Responses ranged from 1 to 7 on a seven-point Likert scale (1=strongly disagree and 7=strongly agree). Scores ranged from 5 to 35, with higher scores indicating a higher level of gratitude.

#### The Positive and Negative Affect Schedule

A total of 20 emotion words, divided into 10 positive affect factors and 10 negative affect ones. It was developed by Watson et al. (1988). Participants must rate the degree to which they endorse each item on a rating scale (1=very slightly or not at all; 5=extremely). Items are divided to create a score for two factors: positive affect and negative affect. Higher scores represent greater endorsement of the construct. Internal consistency was optimal as positive affect depicted an *α*=0.90, and negative affect, *α*=0.91.

### Procedure

This study had the approval from the University ethics committee (number UCV2017-2018-28), in accordance with the Declaration of Helsinki of the World Medical Association.

Participation in this research was voluntary and completely anonymous. A recruitment email was sent via the Prolific platform in the United States. At the beginning of the web-based survey, informed consent information was displayed and therefore accepted by every participant. The questionnaire was available online in May 2020.

### Data Analysis

The analyses were developed through SPSS 22 and Hayes macro for SPSS (2015). After a descriptive approach, assumptions of normality and homogeneity analyses were carried out, prior to further analyses. Secondly, a relational analysis was carried out, as well as linear regression was performed, to make predictions about the variables of interest. Lastly, two moderation analyses were carried out. Regression-based procedures were executed, employing bootstrapping procedures using 10,000 samples (MacKinnon and Fairchild, 2009; Moret-Tatay et al., 2018).

## Results

With regard to CT, 51.6% of the participants reported an upward counterfactual process, reporting that they were *worse*, while 48.4% reported a downward counterfactual process, being *equal or even better*. Descriptive analysis as well as Pearson’s coefficients are depicted in Table 1. A *t*-student test for independent samples was carried out (upward vs. downward) after examining equality of variances which was assumed according to Levene’s test for variances (*p*>0.05). It should be noted that the upward group depicted lower values on positive affect scores and higher values in negative affect scores than the downward group. In terms of gratitude scores, differences did not reach the statistical level between groups. Gratitude did correlate with positive affect in a direct way and vice versa for the negative affect.

**Table 1:** Descriptive statistics on the variables under study using an independent *t*-test across the CT group.

	Group	Mean	SD	*p*	Gratitude	Negative affect	Positive affect
Gratitude	Worse	27.82	5.66	0.14	–		
	Equal or better	28.84	6.26				
Negative affect	Worse	23.24	7.86	<0.001	−0.357^*^	–	
	Equal or better	15.94	7.49	(*d*ʹ=0.94)			
Positive affect	Worse	31.80	8.25	<0.001	0.536^*^	−0.459^*^	–
	Equal or better	36.24	7.18	(*d*ʹ=−0.57)			

Pearson’s correlation coefficients are displayed across the variables of interest.

* *p<0.05*.

Secondly, a lineal regression in the prediction of negative and positive affect scores was carried out. The model was statistically significant for negative affect, as described as follows: *F*_(5,295)_=37.08; MSE=1678.48; *R*^2^=0.38; *p*<0.001. Moreover, the model was also statistically significant for positive affect: *F*_(5,295)_=39.28; MSE=1570.35; *R*^2^=0.40; *p*<0.001. Coefficients are depicted in Table 2. Gender and CT were considered as dummy variables in the model.

**Table 2:** Linear regression coefficients on the prediction of gratitude scores.

Model		B	SE	*β*	*t*	*p*
Negative affect	(Intercept)	43.526	2.499		17.416	0.000
	Age	−0.124	0.025	−0.232	−5.046	0.000
	Gender	0.541	0.787	0.032	0.688	0.492
	Gratitude	−0.228	0.078	−0.161	−2.922	0.004
	CT group	−5.352	0.815	−0.315	−6.567	0.000
	Positive affect	−0.293	0.060	−0.279	−4.924	0.000
Positive affect	(Intercept)	25.152	3.007		8.364	0.000
	Age	−0.050	0.024	−0.098	−2.091	0.037
	Gender	−1.157	0.737	−0.072	−1.570	0.117
	Gratitude	0.599	0.066	0.444	9.121	0.000
	CT group	2.002	0.812	0.124	2.466	0.014
	Negative affect	−0.259	0.053	−0.272	−4.924	0.000

**Figure 1:**
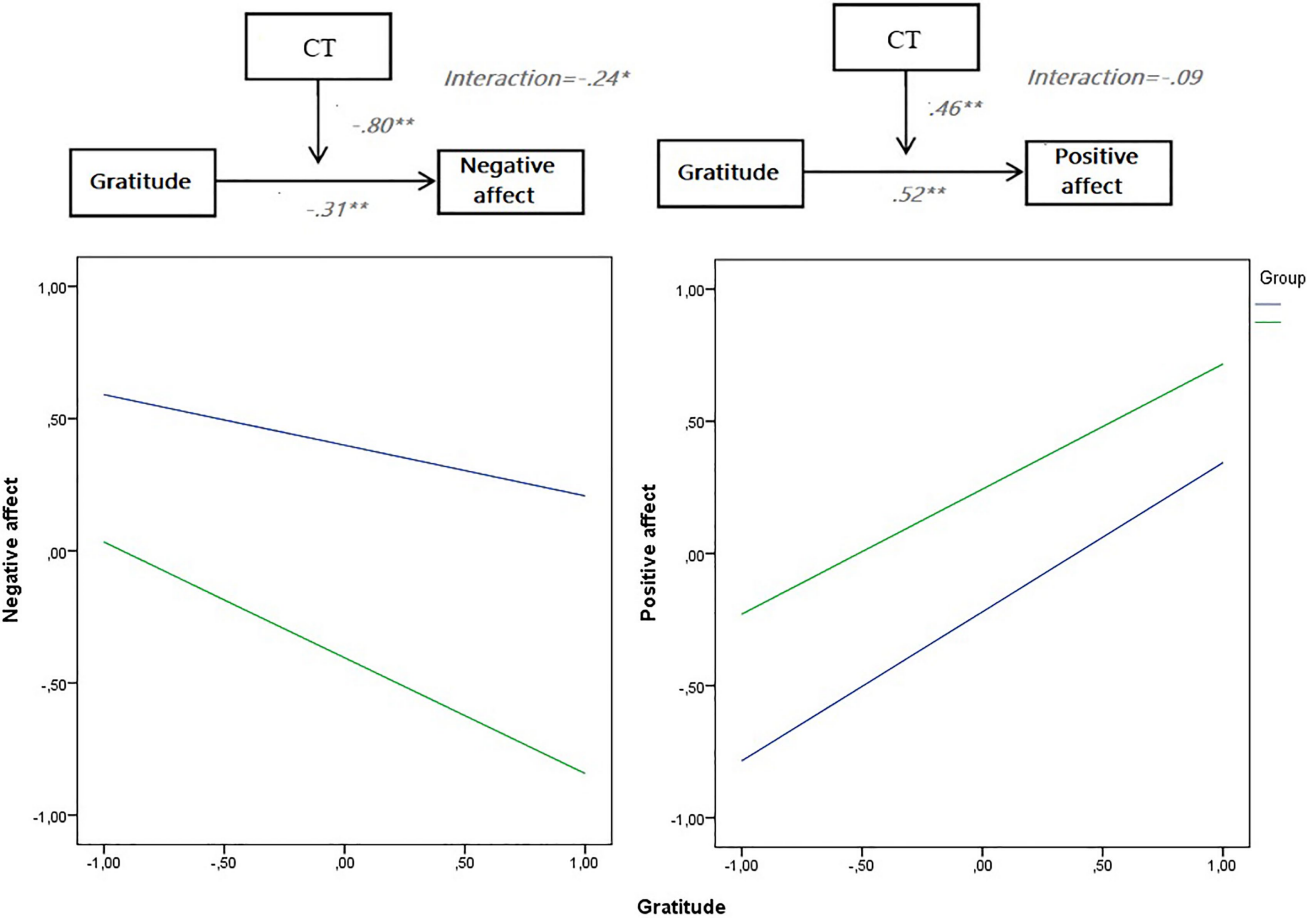
Moderation model for gratitude and positive and negative affect across CT.

Lastly, a moderation model on CT over the relationship between gratitude and positive and negative affect was carried out. Moreover, Figure 1 depicts the proposed models and interactions. While CT did moderate the relationship between gratitude and negative effect, this was not the case for the relationship between gratitude and positive affect. In the first case, the moderation model on negative affect was statistically significant: *F*_(3,300)_=52.24; MSE=0.70; *R*^2^=0.30; *p*<0.001. Coefficients depicted in Figure 1, as well as the interaction, also reached the statistical level. Particularly, the *R*^2^ increase due to the interaction depicted the following values: *R*^2^=0.01; *p*<0.05. On the other hand, the moderation model on positive affect was statistically significant: *F*_(3,300)_=58.62; MSE=0.66; *R*^2^=0.34; *p*<0.001. Even if coefficients depicted in Figure 1 were statistically significant, the interaction was not (*p*=0.29). The conditional effect of gratitude on negative affect at values of the moderator is described in Table 3.

**Table 3:** Conditional effect of X on Y at values of the moderator.

Group measures	Effect	SE	*t*	*p*	LLCI	ULCI
CT groups	−0.19	0.08	−2.33	<0.05	−0.35	−0.02
	−0.43	0.08	−5.39	<0.001	−0.59	−0.27

Effects, standard error (SE), statistical significance, and lower and upper (LLCI and ULCI) level.

## Discussion and Conclusion

The aim of this work was to examine the moderation role of CT on the relationship between gratitude and positive and negative affect. Of note, literature points toward gender differences on age, stress, and psychopathology during pandemics (Barzilay et al., 2020; Benjamin et al., 2020; Pérez et al., 2020; Terry et al., 2020). Thus, these variables were also included in the analysis. The main results of the current study related to the COVID-19 outbreak can be listed as follows: (i) no differences between CT groups in the gratitude trait, but differences in positive and negative affect did occur; (ii) regression models that included age, gratitude, and affect variables predicted negative and positive affects but gender did not reach the statistical level; (iii) two moderation models predicted affect from gratitude, with the CT variable moderating this effect; this moderation effect was also statistically significant in predicting negative affect but it was not statistically significant in predicting positive affect.

The CT variable has been useful to divide the sample into two different groups to address differences in terms of their positive and negative affect, also showing a congruence between measures. In addition, this measure has allowed us to differentiate the counterfactual upward and downward processes, which have also been related to the affects in the direction found in previous literature. In other words, the counterfactual upward process seems to lead to greater negative symptomatology (Broomhall et al., 2017) and the counterfactual downward process to greater wellbeing (e.g., Nicuța and Constantine, 2021). However, this variable did not allow us to find differences in the trait of gratitude suggesting that having high or low levels of gratitude is independent of comparative thinking (where participants indicate that they were worse or, conversely, that they were equal or better). Perhaps having the trait of gratitude does not influence comparative thinking regarding the effects of a pandemic. One should bear in mind that this measurement is a global judgment that does not consider other processes that are involved in the assessment of emotional state. The fact of making a global judgment in relation to CT has implications in the participant’s categorization in relation to gratitude and affect (Bernabe-Valero et al., 2021). In this way, it was found that participants with poorer affect were more biased when making such global judgments, even when reporting similar values in gratitude. Another possible explanation is related to the conceptualization of the trait of gratitude, since the definition of this construct is rather complex. In this research, a unidimensional measure was chosen to make the questionnaires easier for participants but several studies have found differences in results depending whether a unidimensional or multidimensional measure of gratitude has been used (e.g., Martinez-Cortés and Bernabe-Valero, 2018; Bernabé-Valero et al., 2019). Future research should address different gratitude conceptions in its nature (Bernabe-Valero et al., 2020) as well as in its cultural conception (Robustelli and Whisman, 2018). On the other hand, gratitude has been shown to be directly related to positive affect and inversely related to negative affect, as previous literature has shown, particularly in times of a pandemic (Burke et al., 2020; Jiang, 2020) as well as other historical moments (Watkins et al., 2006; Frias et al., 2011). This might indicate that more grateful people may enjoy better affectivity by experiencing higher levels of positive affect and lower levels of negative affect.

Furthermore, these aforementioned variables (CT, gratitude, and affect) plus the age variable, were able to predict negative and also positive affect through two regression models. In relation to age, the general consensus is that older people exhibit a feeling of greater wellbeing and less negative affectivity (e.g., Pinquart, 2001; Ebert et al., 2020). This is congruent with our results which indicate that older people have lower values of negative affectivity. Although the relationship between age and positive affect was statistically significant in the regression model, the value of the slope is very low and therefore not informative. Thus, it can be concluded that negative affect seems to decrease with age which has been considered a compensatory strategy to deal with life losses (Charles et al., 2001; Carstensen et al., 2011; Moreno-Cid et al., 2015). Self-efficacy is also a variable of interest for future lines of research, as it has been escribed to influence positive and negative affect (Bandura et al., 2003). Even if this is not directly considered in the current scope of research, further research in this field might shed light on this triad.

On the other hand, gender did not predict positive and negative affect, which supports the claim of no differences between men and women in terms of COVID-19 affect in the previous literature (Cao et al., 2020; Zhang and Ma, 2020) but not those studies that claimed that women are more negatively affected (Rogowska et al., 2020; Wang et al., 2020). This inconsistency in results alerts us to the need to delve deeper into the processes underlying differences in affective regulation between men and women in order to robustly conclude their affect in the current crisis. Note that this process can be very complex, involving socio-demographic and cultural variables.

As expected, the predictive relationship of gratitude for positive and negative affect has been confirmed. Of interest, the moderation effect occurred for negative affect exclusively. This means that gratitude inversely predicted negative affect experienced in the pandemic, and furthermore, this relationship was moderated by CT, also indicating that this effect was different for participants who used a counterfactual downward process than for those who used a counterfactual upward process. However, gratitude directly predicted positive affect, and this effect was homogeneous for the two groups. Thus, for those participants who indicated that they were *worse*, the protective effect of gratitude on the decrease in negative affectivity decreased, i.e., these effects were moderated; whereas this moderation was less for those who indicated that they were *equal* or *better*.

One mechanism that could explain the gratitude predictive relationship in decreasing negative affect is provided by the broaden-and-build theory (Fredrickson, 2001), which posits that positive emotion experiences broaden people’s momentary thought-action repertoires. In other words, individuals who are more grateful, and, therefore experience higher levels of gratitude in everyday life (McCullough et al., 2004), might broaden their thought-action repertoires due to the positive valence of gratitude. In this way, they may create different thinking and action options in threatening situations. Thereby, emotional management could be improved and negative emotions could be decreased (e.g., scared and nervous emotions as assessed by the positive and negative affect schedule). This mechanism could act in a variety of daily situations and routines, leading to a decrease in negative affect. However, in relation to CT, this comparative thinking is the result of objective conditions derived from the pandemic (e.g., people might have suffered losses in the frequency and quality of social relationships, economic losses, close deaths due to COVID-19, among others), and also have attributed negative meanings to these losses or experienced fears due to the global situation of uncertainty, contributing subjective elements to this judgment. In addition to this, participants engaged in comparative thinking, also bringing into play the counterfactual thinking processes discussed above. We consider that those individuals who in CT indicated that they were “worse” are reflecting the malaise associated with the events that occurred in the pandemic. This malaise would be moderating the beneficial effects of gratitude on negative affect. Thus, the emotion of gratitude would compete with other negative emotions included in their malaise (e.g., for losses during the pandemic). Thus, interfering with the mechanism of amplification of positive emotions, and the beneficial effect of gratitude in decreasing negative affect would be minimized. Conversely, those participants with high levels of CT (equal or better) would be reflecting on the fact that they are not experiencing higher levels of distress than before the pandemic, and, in their case, their dispositional trait of gratitude would be effective in decreasing levels of negative affect, obtaining similar results to studies conducted in non-pandemic times (e.g., for a meta-analysis of the beneficial effects of gratitude on wellbeing, see Portocarrero et al., 2020).

In addition, we consider that the CT could act as an affective schema by integrating experiences and influencing affectivity. Moreover, it has been suggested that the psychological dynamics of gratitude involve a flexible and integrated view for positive and negative aspects of an experience, complemented in a final consideration that privileges a caring and protective global view (Moyano, 2011). In this way, for those participants who considered a more positive general approach with high levels of CT, the moderating effect on affectivity was less accentuated and vice-versa for participants with low levels. However, CT did not have a moderating effect between gratitude and positive affect, indicating that grateful people (regardless of their CT score) will have higher levels of positive affect and less grateful people will have lower levels of positive affect. Unlike the previous moderation model, CT-associated malaise (e.g., worse) does not diminish the predictive relationship of gratitude with positive affect, also revealing that positive emotions are more robustly related to each other than positive emotions (e.g., daily gratitude) and negative affect, as this relationship may be moderated by other variables such as CT. This result seems to reinforce the idea of distinct underlying patterns for positive and negative affect (i.e., Fredrickson and Joiner, 2002).

An additional aspect to consider is the instrument employed for the assessment of gratitude based on a unidimensional scale in the current study, which has not allowed us to assess aspects as interesting as gratitude in terms of suffering, but are included in other scales underling the term of gratitude (e.g., the G20 in Bernabé-Valero et al., 2014; English adaptation in Bernabe-Valero et al., 2020). In other words, this seems particularly suitable for assessing whether people can feel gratitude in situations that generate suffering, such as the ongoing crisis resulting from the current pandemic. We suggest that the inclusion of this scale in a predictive model could have promising results in the moderating effect of CT between gratitude and affectivity, using longitudinal designs that might capture the experience of gratitude in adverse situations once these have ended.

Definitely, these models might not only help to clarify the relationships between dispositional traits and affect, in line with other studies in the field (McCullough et al., 2004; Wood et al., 2008), but could also include comparative thinking variables from the cognitive domain, showing that these variables can modify these relationships. In this way, the relevance of multivariate models that include different affective, cognitive, and dispositional dimensions is emphasized.

The current research has also explored the prediction of negative and positive affect in times of COVID, based on different dispositional, demographic, and cognitive variables. In addition, these results contribute to provide more evidence on the importance of gratitude as a human strength that promotes benefits in psychological health. Taken into consideration that individuals with higher scores in negative affect tend to have a higher incidence of depressive symptoms compared to the general population (Roberts and Kassel, 1996), these results may have therapeutic and educational implications for health programs. These might be of interest for the improvement of the affective experience, especially in times such as the COVID-19 pandemic. In light of the results obtained, we consider that they might provide clues that go beyond this adverse situation to come across different basic psychological needs and processes that explain human behavior.

The main limitations of this study can be described as follows: (i) the sample was selected through an incidental sampling; (ii) data were collected in a self-report way; (iii) data on COVID-19 knowledge or exposure were not collected. Future lines of research should include more information on knowledge regarding COVID-19 and exposure, to better understand the role of positive and negative affect. Nevertheless, it is expected that these results are of interest at both applied and theoretical levels.

## Data Availability Statement

The raw data supporting the conclusions of this article will be made available by the authors, without undue reservation.

## Ethics Statement

The studies involving human participants were reviewed and approved by Universidad Católica de Valencia San Vicente Mártir committee (number UCV2017-2018-28). The patients/participants provided their written informed consent to participate in this study.

## Author Contributions

GB-V conceived the presented idea. GB-V, CM-T, JB-M, and RA-M developed the theory and performed the computations. All authors discussed the results and contributed to the final manuscript.

## Conflict of Interest

The authors declare that the research was conducted in the absence of any commercial or financial relationships that could be construed as a potential conflict of interest.

## Publisher’s Note

All claims expressed in this article are solely those of the authors and do not necessarily represent those of their affiliated organizations, or those of the publisher, the editors and the reviewers. Any product that may be evaluated in this article, or claim that may be made by its manufacturer, is not guaranteed or endorsed by the publisher.
